# Targeted Mutation of a Non-catalytic Gating Residue Increases the Rate of *Pseudomonas aeruginosa*d-Arginine Dehydrogenase Catalytic Turnover

**DOI:** 10.1021/acs.jafc.3c05328

**Published:** 2023-11-07

**Authors:** Joanna Afokai Quaye, Daniel Ouedraogo, Giovanni Gadda

**Affiliations:** † Department of Chemistry, Georgia State University, Atlanta, Georgia 30302-3965, United States; ‡ Department of Biology, Georgia State University, Atlanta, Georgia 30302-3965, United States; § Center for Diagnostics and Therapeutics, Georgia State University, Atlanta, Georgia 30302-3965, United States

**Keywords:** *Pseudomonas aeruginosa*d-arginine dehydrogenase, enzyme turnover, product release, hydride transfer, loop dynamics

## Abstract

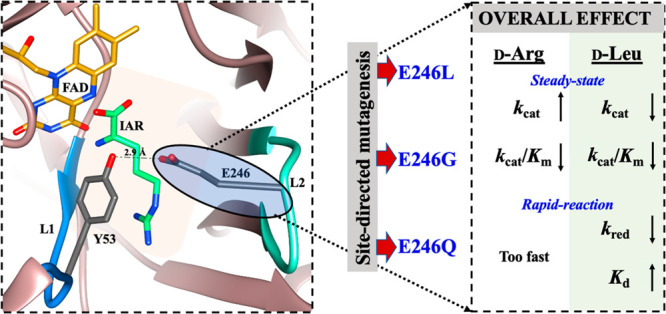

Commercial food and l-amino acid industries rely on bioengineered d-amino acid oxidizing enzymes to detect and remove d-amino acid contaminants. However, the bioengineering of enzymes to generate faster biological catalysts has proven difficult as a result of the failure to target specific kinetic steps that limit enzyme turnover, *k*_cat_, and the poor understanding of loop dynamics critical for catalysis. *Pseudomonas aeruginosa*d-arginine dehydrogenase (*Pa*DADH) oxidizes most d-amino acids and is a good candidate for application in the l-amino acid and food industries. The side chain of the loop L2 E^246^ residue located at the entrance of the *Pa*DADH active site pocket potentially favors the closed active site conformation and secures the substrate upon binding. This study used site-directed mutagenesis, steady-state, and rapid reaction kinetics to generate the glutamine, glycine, and leucine variants and investigate whether increasing the rate of product release could translate to an increased enzyme turnover rate. Upon E^246^ mutation to glycine, there was an increased rate of d-arginine turnover *k*_cat_ from 122 to 500 s^–1^. Likewise, the *k*_cat_ values increased 2-fold for the glutamine or leucine variants. Thus, we have engineered a faster biocatalyst for industrial applications by selectively increasing the rate of the *Pa*DADH product release.

## Introduction

Amino acids are essential for the survival of all living organisms. Of the two classifications of amino acids, the l-amino acid isomers form the core of proteins, such as enzymes, hormones, receptors, and antibodies, and the d-amino acid isomers participate in numerous physiological processes, like the neurotransmission by d-serine and d-aspartate.^1−3^ There were no detection methods for d-amino acids for several decades before improved analytical techniques, such as gas chromatography, high-performance liquid chromatography, and chiral columns, were introduced in the 1990s.^1−4^ Recently, d-amino acid detection has become significantly important in the food industry as a result of the heat-induced spontaneous racemization of l-amino acids to generate the d-isomers that contaminate food preparations.^5,6^ Current commercial methods to synthesize l-amino acids depend upon the design and engineering of d-amino acid oxidizing enzymes through directed evolution and rational design to detect d-amino acids and resolve racemic amino acid mixtures.^7,8^ Such engineered enzymes rapidly accomplish d-amino acid detection with low costs, high sensitivity, and high accuracy compared to classical analytical techniques, like high-performance liquid chromatography.^8^ However, the current commercial methods rely on a limited understanding of enzyme catalysis, which is crucial for developing effective and improved biocatalysts.

Enzymes are highly efficient macromolecules that catalyze many biochemical reactions in living cells.^9−13^ Enzymes assume multiple conformational changes during catalysis and can accelerate reactions by many orders of magnitude.^12−18^ During enzyme engineering, scientists often aim to increase the overall rate of enzyme turnover, *k*_cat_, which, unless demonstrated otherwise, might be limited by the chemical step of catalysis and the mechanical step of product release enhanced by favorable enzyme motions.^19−21^ However, there is a tendency for engineers to consider the *k*_cat_ parameter as a single kinetic step, which may limit the approaches used in protein engineering. Scientists do not fully understand the effects of enzyme motions on enzyme catalysis, such as loop dynamics. Hence, engineered proteins often exhibit poor catalytic abilities compared to their native proteins.^22−25^ Most enzymes undergo natural evolutionary processes that fashion them for better functionality in an organism, including mutations into more active forms that result in improved enzyme flexibility for catalysis.^9−11,26^ For protein engineering for therapeutics, such as in treating cardiovascular diseases and cancer therapy,^27−29^ and for applications in the food industry, studies on flexible enzyme dynamics are also necessary to generate better biocatalysts.

Loops, the most flexible secondary structural elements of enzymes, play significant roles in enzyme catalysis. Loops mediate substrate binding, maintain the integrity of the active site, protect from the bulk solvent after enzyme–substrate complex formation, and initiate conformational changes.^30−33^ Multiple studies demonstrate how loop modulations can alter the catalytic function of an enzyme.^20,34−36^ In a site-directed mutagenesis study that investigates the roles of loop residues A^85^ and I^86^ located at the binding pocket of *Thermoanaerobacter brockii* alcohol dehydrogenase, the engineered enzyme variants A^85^G and I^86^L gained the ability to reduce bulky ketones to the corresponding alcohols with high enantioselectivities.^37^ Similarly, a study on cumene dioxygenase demonstrates how site-directed mutageneses of multiple critical active site loop residues resulted in significant improvement of product formation with alterations in the regioselectivity and enantioselectivity of the enzyme.^33^ In cytochrome P450, it was observed that a single mutation in the P450 F/G loop could cause a regioselectivity switch.^38^ These studies demonstrate that loop residues are essential for enzyme catalysis. However, there are only few studies on increased enzyme turnover rates resulting from the mutation of loop residues as reported for *Bacillus subtilis* xylanase and human kynureninase.^39,40^ Perhaps the failure to produce more engineered enzymes with better catalytic turnovers compared to the native proteins lies in the inability of scientists to design mutations that distinctively improve either of the two kinetic steps that usually limit the overall rate of enzyme turnover, *k*_cat_: catalysis and product release.

**Scheme 1:**
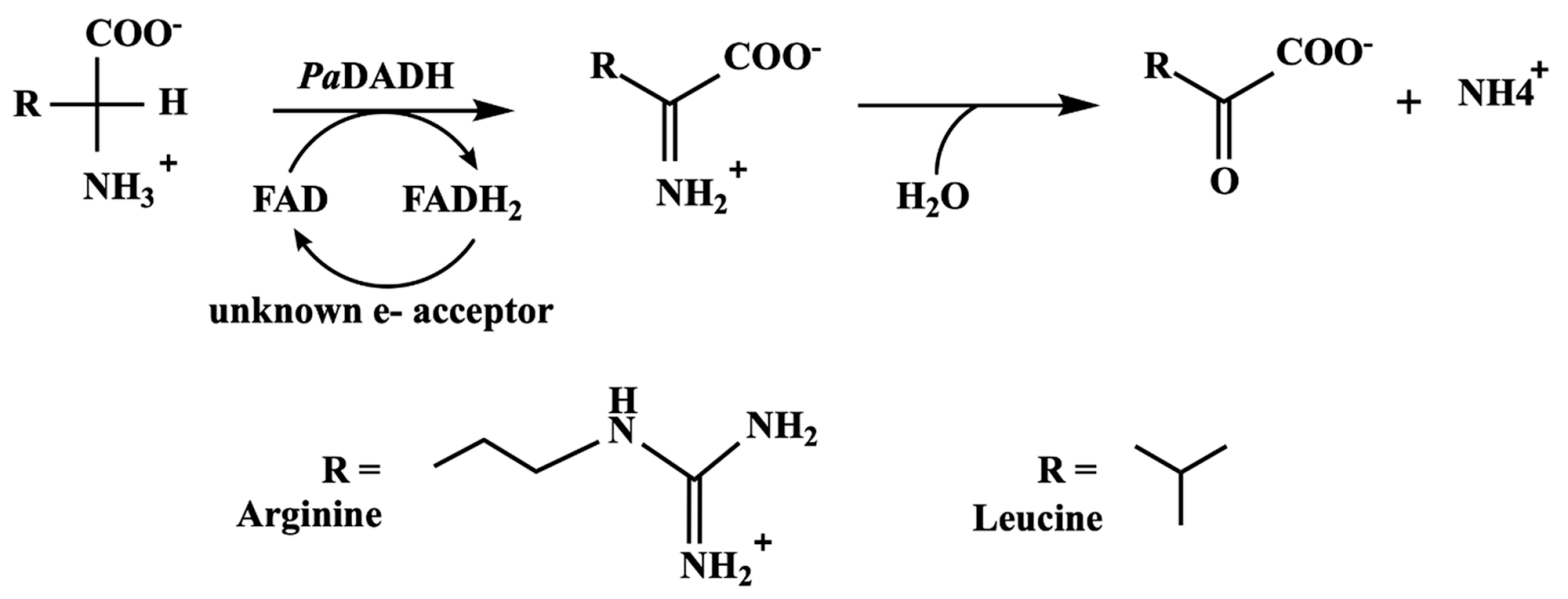
General Reaction Scheme of *P. aeruginosa*d-Arginine Dehydrogenase

**Figure 1:**
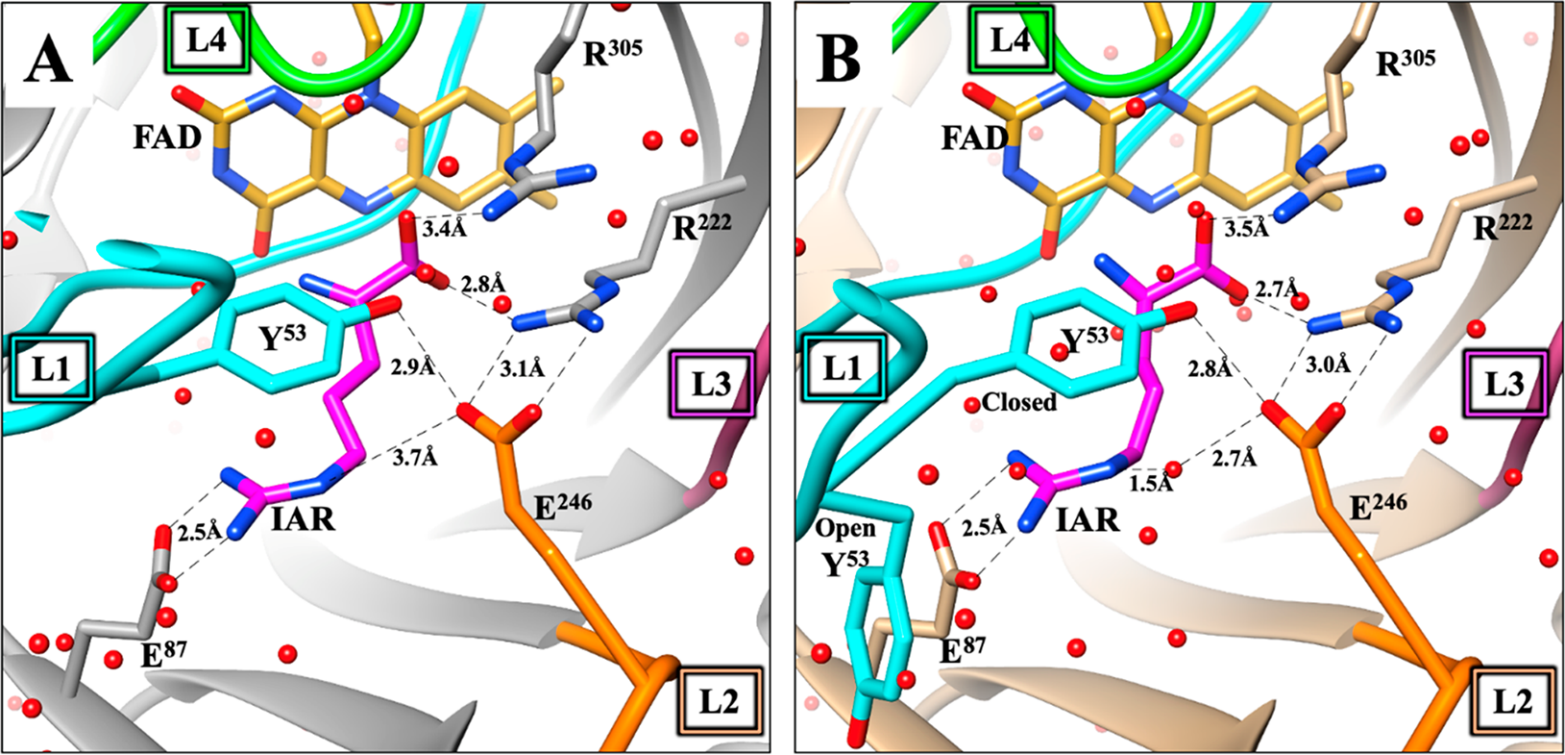
Interactions between E^246^, Y^53^ gate, and active site residues in *Pa*DADH. (A) Crystal structure of *Pa*DADH in complex with iminoarginine (3NYE). (B) Crystal structure of free *Pa*DADH with 30% iminoarginine occupancy (3NYC).^41,44^ E^246^ is found in coral. Y^53^ is in cyan. N atoms are shown in blue, and O atoms are shown in red. The FAD cofactor is represented by its isoalloxazine ring, with the C atoms in gold. IAR represents the iminoarginine product as presented in magenta. Loops are shown in cyan (L1), coral (L2), purple (L3), and green (L4). Hydrogen bond interactions are shown as dashed lines. The PDB files 3NYE and 3NYC were visualized and analyzed using the UCSF Chimera software.^49^

*Pseudomonas aeruginosa*d-arginine dehydrogenase (*Pa*DADH) is a flavin-dependent enzyme with broad substrate specificity.^41−43^ The enzyme oxidizes all d-amino acids with proteinaceous l-amino acid counterparts to their corresponding α-keto acids and ammonia (Scheme 1), except d-aspartate and d-glutamate,^44^ making *Pa*DADH a good candidate for applications in the l-amino acid industry, where pure l-amino acids are synthesized for various applications. During the ping-pong bi-bi steady-state catalytic cycle of the enzyme, substrate deprotonation occurs by the direct release of the α-amino proton to the solvent without the involvement of protein residues,^42,45,46^ making *Pa*DADH similar to d-amino acid oxidase (DAAO) in its catalytic mechanism.^47^ DAAO displays a higher substrate specificity toward neutral d-amino acid substrates.^48^ The highest *k*_cat_/*K*_m_ values for *Pa*DADH were observed for cationic side-chain substrates, d-arginine and d-lysine, with values of ∼10^6^ M^–1^ s^–1^, making *Pa*DADH the only one among amine-oxidizing enzymes with a high *k*_cat_/*K*_m_ value toward a cationic d-amino acid isomer.^41−43,48^ The *k*_cat_ parameter of *Pa*DADH with d-arginine as a substrate has been demonstrated to be limited by both hydride transfer and product release.^42^ Recent studies on *Pa*DADH show four active site loops in the crystal structure of *Pa*DADH (loops L1–L4).^19,41,44^ Residue Y^53^ of loop L1 acts as a gate that adopts an open conformation to allow for substrate binding and product egress. Upon substrate binding, the Y^53^ gate adopts a closed conformation to secure the substrate for catalysis.^41,44^ The I^335^ residue of loop L4 has been demonstrated to have a long-range dynamic effect that regulates the access and exit of ligands, leading to the accumulation of the ES and EP complexes.^19^ Although the catalytic roles of the residues in loops L2 and L3 of *Pa*DADH have not been explored, loop L2 is located at the active site entrance and likely narrows the entrance of the active site pocket.^44^ Structural analyses of the ligand-bound and -free (with 30% occupancy of ligand) forms of the enzyme reveal that the position of the E^246^ residue remains unaltered upon ligand binding. As shown in the X-ray crystal structure of the enzyme in complex with the iminoarginine product of d-arginine oxidation (Figure 1), the E^246^ residue of loop L2 points toward the active site and likely interacts with the active site residue R^222^ and the Y^53^ gate.^41,44^ Additionally, from the two crystal structures of *Pa*DADH in complex with iminoarginine, the E246 residue likely interacts with the guanidinium moiety of iminoarginine, either directly (Figure 1A) or through a water-meditated interaction (Figure 1B).^41,44^ Thus, we propose that the E^246^ residue of loop L2 participates in *Pa*DADH catalysis by mediating the steps of substrate binding and product release from the active site.

This study used site-directed mutagenesis to replace the E^246^ residue in loop L2 with glutamine, glycine, or leucine, yielding the respective E^246^Q, E^246^G, or E^246^L variant enzymes to investigate whether selectively increasing the rate of product release without altering catalysis could translate into an increased rate of enzyme turnover. The variant enzymes have been studied and characterized in their kinetic properties using steady-state and rapid reaction kinetic approaches to elucidate the role of the loop L2 E^246^ residue in *Pa*DADH catalysis.

## Experimental Procedures

### Materials

*Escherichia coli* strain Rosetta (DE3) pLysS and the pET20b(+) expression vector were purchased from Novagen (Madison, WI, U.S.A.). DH5α *E. coli* strain was purchased from Life Technologies, Inc. The QIAprep Spin Miniprep Kit and QIAquick polymerase chain reaction (PCR) purification kit were obtained from Qiagen (Valencia, CA, U.S.A.). *Pfu* DNA polymerase was purchased from Stratagene (La Jolla, CA, U.S.A.). The enzymes *Dpn*I, calf intestinal alkaline phosphatase, and T4 DNA ligase were purchased from New England Biolabs (Ipswich, MA, U.S.A.). Oligonucleotides for site-directed mutagenesis and sequencing of the variant genes were purchased from Sigma Genosys (The Woodlands, TX, U.S.A.). A HiTrap chelating HP 5 mL affinity column was from GE Healthcare, and isopropyl 1-thio-d-galactopyranoside was from Promega. Phenazine methosulfate (PMS) was obtained from Sigma-Aldrich (St. Louis, MO, U.S.A.). d-Amino acids were obtained from Alfa-Aesar (Ward Hill, MA, U.S.A.). All other reagents used were obtained with the highest purity commercially available.

### Site-Directed Mutagenesis, Protein Expression, and Purification

To investigate the role of the E^246^ residue of *Pa*DADH, the glutamate 246 to glutamine mutant (E^246^Q), glutamate 246 to glycine mutant (E^246^G), and glutamate 246 to leucine mutant (E^246^L) variant genes of *Pa*DADH were generated by mutagenic PCR with the pET20b(+)/PA3863 plasmid harboring the wild-type gene (*dau*A) as a template and mutagenic primers containing the corresponding site mutations. A concentration of 5% dimethyl sulfoxide was added to the PCR reaction mixture to ensure proper separation of the highly GC-rich, double-stranded DNA template. Site-directed mutagenesis amplicons were purified according to the instructions of the manufacturer using the QIAquick PCR purification kit. The purified samples were treated with *Dpn*I at 37 °C for 2 h and transformed into *E. coli* strain DH5α cells. Each mutation was confirmed by sequencing the gene using Humanizing Genomics Microgen USA Corp. in Maryland. The E^246^Q, E^246^G, and E^246^L variant enzymes of *Pa*DADH were expressed in *E. coli* Rosetta (DE3) pLysS cells and purified to homogeneity using the classical purification method as previously described for the *Pa*DADH wild-type enzyme in the presence of 10% (v/v) glycerol to minimize enzyme instability and to prevent the loss of the FAD cofactor from the enzyme.^26^ The enzymes were stored at −20 °C in 20 mM Tris–Cl at pH 8.0 and 10% glycerol and were found to be active for at least 6 months.

### Steady-State Kinetic Investigation of the *Pa*DADH Variant Enzymes

To investigate the role of the E^246^ residue in the steady-state kinetics of *Pa*DADH, the initial rates were measured to determine the steady-state kinetic parameters of the E^246^Q, E^246^G, or E^246^L variant enzyme with d-arginine as a substrate and PMS as an electron acceptor at pH 10.0.^42,46^*Pa*DADH is a true dehydrogenase, and as such, it does not react with molecular oxygen;^42^ thus, the spontaneous reduction of molecular oxygen by the *Pa*DADH reduced PMS was followed with a Clark-type oxygen electrode. The final enzyme concentrations in 1 mL reaction mixtures ranged from 7 to 70 nM, and those of d-arginine ranged from 0.04 to 1.5 mM for all enzyme variants and from 0.01 to 0.5 mM for the wild-type enzyme. In all enzymatic assays, the *K*_m_ value was within the range of the substrate concentrations used. The enzymatic assays were carried out at 25 °C in 20 mM sodium pyrophosphate at pH 10.0. Substrate solutions were prepared in sodium pyrophosphate buffer, and the pH values were readjusted after the amino acid substrates were dissolved. The concentration of PMS was fixed at 1 mM because the *K*_m_ value for PMS in the *Pa*DADH wild type was previously determined to be ∼10 μM, irrespective of the substrate used.^26^ To ensure that the variant enzymes were fully saturated with PMS, the steady-state kinetic parameters were also determined at 1.5 mM PMS and similar results were obtained. In a control experiment to test the hypothesis of the interaction of residue 246 with the guanidinium moiety of the iminoarginine product, the same conditions described above were repeated with d-leucine as a substrate. d-Leucine concentrations ranged from 0.8 to 50 mM.

Under the same steady-state conditions described above for all variant enzymes with d-arginine or d-leucine as the substrate, all enzymes had a negligible oxidase activity of ∼0.2 s^–1^ without PMS. Hence, the steady-state kinetic parameters with PMS report only on the dehydrogenase activity of the enzymes.

### Reductive Half-Reaction

To determine the *K*_d_ values for d-leucine with the recombinantly expressed *Pa*DADH E^246^Q, E^246^G, and E^246^L variant enzymes, the reduction of the enzyme-bound flavin was followed by monitoring the decrease in absorbance at 446 nm upon mixing the variant enzymes with varying concentrations of the reducing substrate. The time-resolved absorbance spectroscopies of the reduction of the *Pa*DADH E^246^Q, E^246^G, and E^246^L variant enzymes with d-leucine were carried out with a SF-61DX2 Hi-Tech KinetAsyst high-performance stopped-flow spectrophotometer equipped with a photomultiplier detector and thermostated with a water bath at 25 °C under anaerobic conditions. Anaerobiosis of the stopped-flow spectrophotometer was carried out by overnight incubation of a solution containing 5 mM glucose and 1 μM glucose oxidase in 100 mM sodium pyrophosphate at pH 6.0 and room temperature. All enzyme variants were passed through a desalting PD-10 column equilibrated with 20 mM sodium pyrophosphate at pH 10.0 and transferred to a tonometer, which was made anaerobic by alternating flushing with argon and degassing by applying a vacuum for 20 cycles. The different substrate solutions were loaded into syringes and flushed for 30 min with argon before being mounted onto the stopped-flow instrument. Moreover, 2 mM glucose and 0.5 μM glucose oxidase were present in all buffers, enzyme solutions, and substrate solutions to completely remove traces of oxygen.

The reductive half-reaction for all variant enzymes was performed under pseudo-first-order conditions, where the enzyme concentration after mixing with the substrate was ∼10 μM and that of d-leucine was between 1 and 40 mM. Equal volumes of the enzyme and d-leucine were mixed in the stopped-flow spectrophotometer in single-mixing mode following established procedures with an instrument dead time of 2.2 ms.

With all variant enzymes, flavin reduction occurred almost fully within the mixing time of the stopped-flow spectrophotometer when d-arginine was used as the substrate at 25 °C, as previously established for *Pa*DADH wild type, preventing the determination of the rapid reaction parameters of the variant enzymes with d-arginine as the substrate at 25 °C.

### Data Analysis

All kinetic data were fit with KaleidaGraph software (Synergy Software, Reading, PA, U.S.A.). The steady-state kinetic parameters determined at varying concentrations of d-arginine and d-leucine as substrates and fixed concentrations of PMS were determined using the Michaelis–Menten equation for a single substrate.

The time-resolved stopped-flow traces from the flavin reductions of the *Pa*DADH E^246^Q, E^246^G, and E^246^L variant enzymes were fit to eq 1 using KinetAsyst 3 (TgK-Scientific, Bradford on-Avon, U.K.) software. The equation describes a single-exponential process, in which *A* represents the absorbance at 446 nm at time *t*, *B* represents the amplitude of the decrease in absorbance, *k*_obs_ defines the observed rate constants for the change in absorbance associated with flavin reduction, and *C* is an offset value accounting for the non-zero absorbance of the fully reduced enzyme-bound flavin at infinite time.
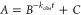
1The concentration dependence of the observed rate constants for flavin reduction was analyzed with eq 2, which describes a hyperbolic trend with d-leucine with a *y*-intercept value of 0. Using an equation that defines a hyperbolic saturation with a finite *y*-intercept yielded a value not significantly different from 0 for the *y*-intercept. In eq 2, *k*_obs_ represents the observed first-order rate constant for the reduction of enzyme-bound flavin at any given substrate concentration, *S* represents the concentration of the amino acid substrate, *k*_red_ is the limiting first-order rate constant for the enzyme-bound flavin reduction at saturating substrate concentrations, and *K*_d_ is the apparent equilibrium constant for the dissociation of the enzyme–substrate complex into the free substrate and enzyme.
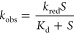
2

## Results

### Steady-State Kinetics of *Pa*DADH E^246^ Variants with d-Arginine and d-Leucine as Substrates

To characterize the *Pa*DADH E^246^ variants E^246^Q, E^246^G, and E^246^L in their steady-state kinetic properties and to understand the role of residue E^246^ in *Pa*DADH substrate capture and catalysis, the apparent steady-state kinetic parameters of all enzyme variants were determined with varying concentrations of the amino acid substrate and a fixed saturating concentration of 1 mM PMS as an electron acceptor. The data for all enzyme variants were compared to that of the wild-type enzyme as a reference. Initial reaction rates were monitored using a Clark-type oxygen electrode monitoring the PMS-driven oxygen consumption reporting on enzyme turnover in 20 mM NaPP_i_ at pH 10.0 and 25 °C. The data were fit to the Michaelis–Menten equation, yielding the steady-state kinetic parameters shown in Tables 1 and 2.

**Table 1:** Steady-State Kinetic Parameters of the *Pa*DADH E^246^ Variants and Wild Type with d-Arginine^a^

enzyme	*k*_cat_/*K*_m_ (M^–1^ s^–1^)	*K*_m_ (mM)	*k*_cat_ (s^–1^)
E246Q	1500000 ± 50000	0.19 ± 0.01	275 ± 3
E246G	2500000 ± 200000	0.20 ± 0.02	500 ± 20
E246L	871000 ± 35000	0.30 ± 0.02	265 ± 5
wild type	3600000 ± 200000	0.033 ± 0.002	122 ± 2

a Steady-state enzyme activities were measured at varying concentrations of d-arginine and fixed 1 mM PMS concentration. All assays were carried out in 20 mM NaPP_i_ at pH 10.0 and 25 °C.

**Table 2:** Steady-State and Rapid Reaction Kinetic Parameters of the *Pa*DADH E^246^ Variants and Wild Type with d-Leucine^a^

enzyme	*k*_cat_/*K*_m_ (M^–1^ s^–1^)	*K*_m_ (mM)	*k*_cat_ (s^–1^)	*K*_d_ (mM)	*k*_red_ (s^–1^)
E246Q	2600 ± 160	11 ± 1	30 ± 1	11 ± 1	48 ± 1
E246G	4800 ± 340	7.3 ± 0.7	35 ± 1	11.0 ± 0.3	43 ± 4
E246L	4600 ± 340	5.8 ± 0.6	27 ± 1	12 ± 1	35 ± 1
wild type	11000 ± 880	6.6 ± 0.5	73 ± 3	6.8 ± 0.2^b^	91 ± 3^b^

a Steady-state enzyme activities were measured at varying concentrations of d-leucine and fixed 1 mM PMS concentration. Rapid reaction kinetics were measured at varying concentrations of d-leucine under anaerobic conditions. All assays were carried out in 20 mM NaPP_i_ at pH 10.0 and 25 °C.

b Data for the wild-type enzyme were as previously reported.^46^

When d-arginine was used as a substrate with the E^246^G variant, there was a 4-fold increase in the *k*_cat_ value compared to the wild type. The *K*_m_ value increased by 7-fold, while the *k*_cat_/*K*_m_ value decreased by 1.5-fold. The data for the E^246^Q variant followed similar trends with a 2-fold increase in the value for the *k*_cat_ parameter, a 7-fold increase in the value for the *K*_m_ parameter, and a 2.4-fold decrease in the value for the *k*_cat_/*K*_m_ parameter compared to the wild type. Similarly, there was a 2-fold increase in the value for the *k*_cat_ parameter, a 10-fold increase in the value for the *K*_m_ parameter, and a 4-fold decrease in the value for the *k*_cat_/*K*_m_ parameter when the E^246^L variant was tested with d-arginine, as shown in Table 1.

In the control experiment, when the substrate was changed to the smaller d-leucine substrate, the *k*_cat_/*K*_m_ values decreased to similar extents for all enzymes, as observed with the d-arginine substrate. In contrast, with d-leucine, the *k*_cat_ values decreased by 2–3-fold for all enzyme variants compared to the wild-type enzyme, while the values for the *K*_m_ values were not significantly different from that of the wild-type enzyme, as shown in Table 2.

### Rapid Reaction Kinetics of *Pa*DADH E^246^ Variants with d-Leucine as the Substrate

**Figure 2:**
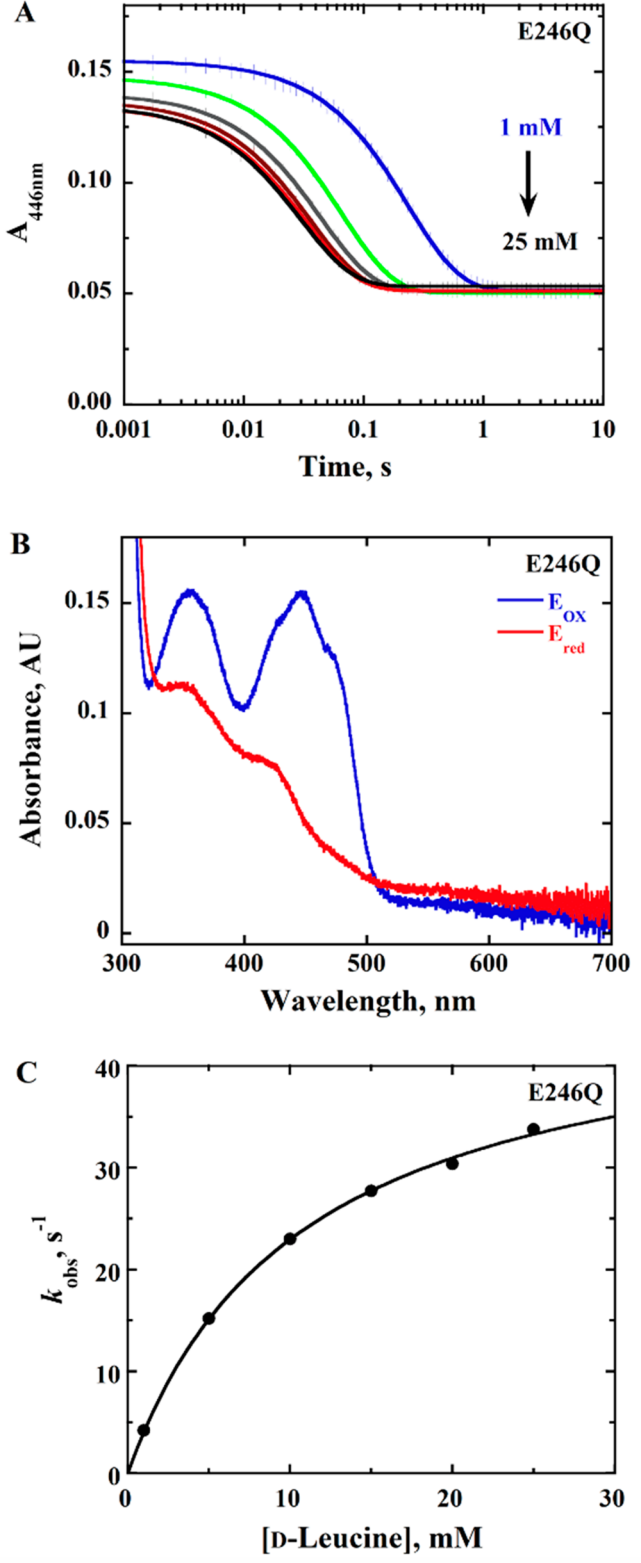
Anaerobic reduction of *Pa*DADH E246Q with d-leucine as the substrate. (A) Stopped-flow traces of *Pa*DADH E246Q at 446 nm with varying concentrations of d-leucine (1–25 mM) fit with eq 1. In the interest of clarity, 1 out of every 10 experimental points is shown (vertical lines). Each trace is the average of triplicate runs at each substrate concentration. Note the log time scale. (B) Absorption spectra of *Pa*DADH E246Q showing fully oxidized flavin before reduction (blue trace) and fully reduced flavin after reduction with 25 mM d-leucine (red trace). (C) Concentration dependence of d-leucine on *k*_obs1_ with *Pa*DADH E246Q fit with eq 2. The single point shown at each substrate concentration is the *k*_obs_ value obtained from the fit of the average of triplicate runs with eq 1, yielding an error of ≤5%. The assay was performed in 20 mM NaPP_i_ at pH 10.0 using a SF-61DX2 Hi-Tech KinetAsyst high-performance stopped-flow spectrophotometer thermostated at 25 °C and equipped with a photomultiplier detector under anaerobic conditions. The instrumental dead time is 2.2 ms.

**Figure 3:**
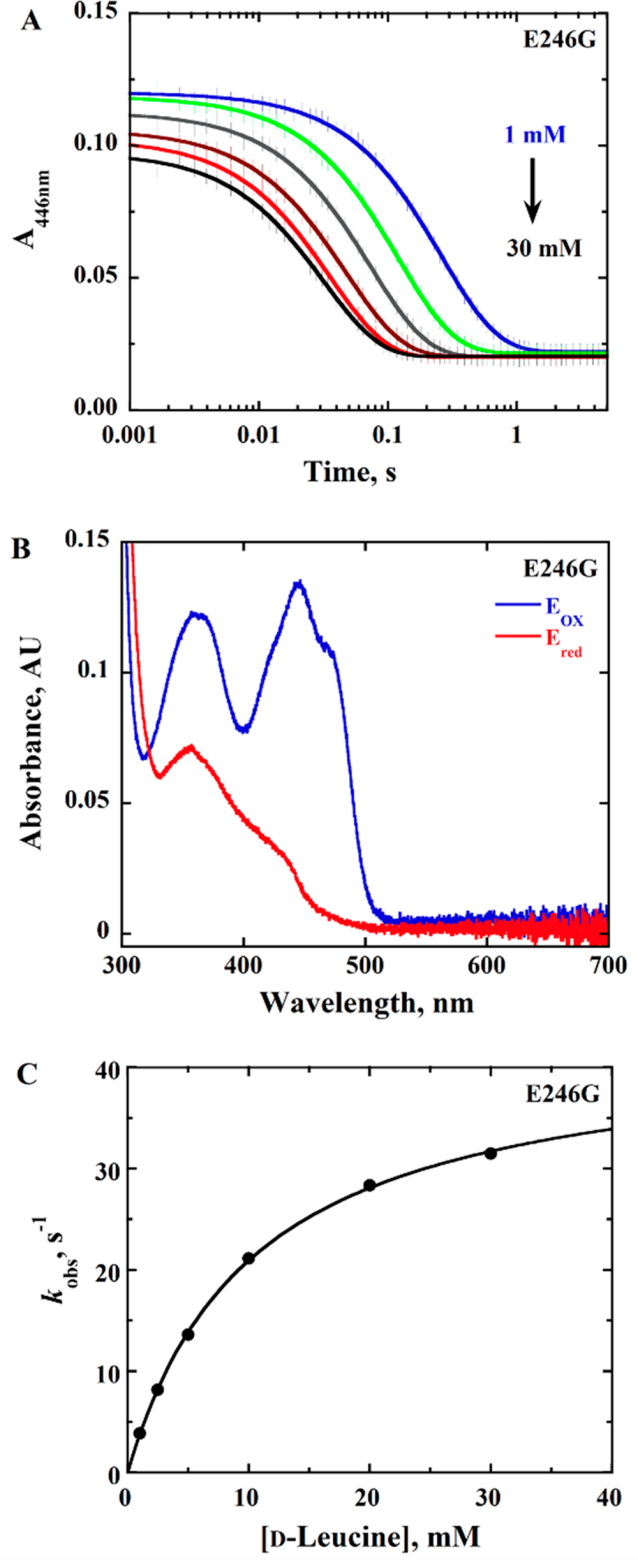
Anaerobic reduction of *Pa*DADH E246G with d-leucine as the substrate. (A) Stopped-flow traces of *Pa*DADH E246G at 446 nm with varying concentrations of d-leucine (1–30 mM) fit with eq 1. In the interest of clarity, 1 out of every 10 experimental points is shown (vertical lines). Each trace is the average of triplicate runs at each substrate concentration. Note the log time scale. (B) Absorption spectra of *Pa*DADH E246G showing fully oxidized flavin before reduction (blue trace) and fully reduced flavin after reduction with 30 mM d-leucine (red trace). (C) Concentration dependence of d-leucine on *k*_obs1_ with *Pa*DADH E246G fit with eq 2. The single point shown at each substrate concentration is the *k*_obs_ value obtained from the fit of the average of triplicate runs with eq 1, yielding an error of ≤7%. The assay was performed in 20 mM NaPP_i_ at pH 10.0 using a SF-61DX2 Hi-Tech KinetAsyst high-performance stopped-flow spectrophotometer thermostated at 25 °C and equipped with a photomultiplier detector under anaerobic conditions. The instrumental dead time is 2.2 ms.

**Figure 4:**
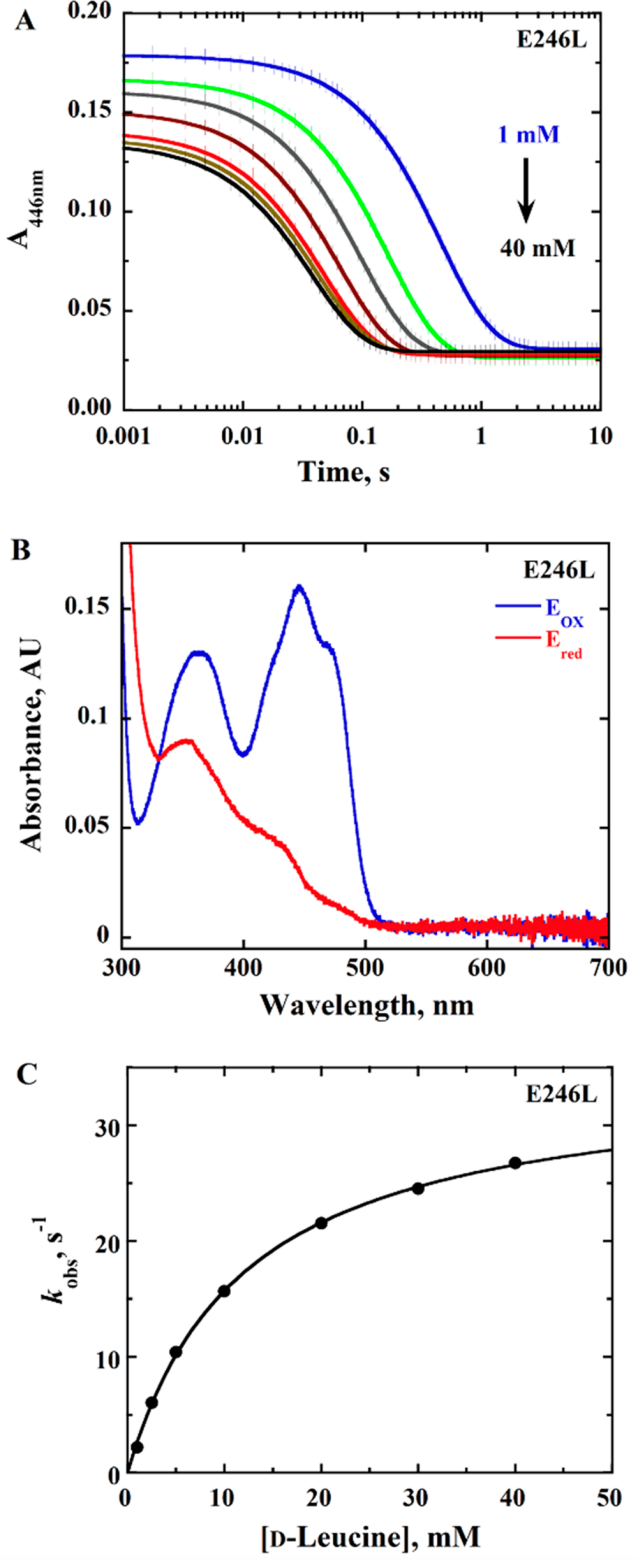
Anaerobic reduction of *Pa*DADH E246L with d-leucine as the substrate. (A) Stopped-flow traces of *Pa*DADH E246L at 446 nm with varying concentrations of d-leucine (1–40 mM) fit with eq 1. In the interest of clarity, 1 out of every 10 experimental points is shown (vertical lines). Each trace is the average of triplicate runs at each substrate concentration. Note the log time scale. (B) Absorption spectra of *Pa*DADH E246L showing fully oxidized flavin before reduction (blue trace) and fully reduced flavin after reduction with 40 mM d-leucine (red trace). (C) Concentration dependence of d-leucine on *k*_obs1_ with *Pa*DADH E246L fit with eq 2. The single point shown at each substrate concentration is the *k*_obs_ value obtained from the fit of the average of triplicate runs with eq 1, yielding an error of ≤6%. The assay was performed in 20 mM NaPP_i_ at pH 10.0 using a SF-61DX2 Hi-Tech KinetAsyst high-performance stopped-flow spectrophotometer thermostated at 25 °C and equipped with a photomultiplier detector under anaerobic conditions. The instrumental dead time is 2.2 ms.

The time-resolved anaerobic reduction of the *Pa*DADH E^246^ variant enzymes was investigated to gain insight into the role of E^246^ in flavin reduction using a stopped-flow spectrophotometer by monitoring the loss of absorbance of the oxidized flavin at 446 nm upon mixing the enzyme with d-leucine at pH 10.0 and 25 °C. For all variant enzymes, a full reduction of enzyme-bound flavin was observed (Figures 2–4). Pseudo-first-order conditions with 10 μM enzyme and 1–40 mM d-leucine were maintained, and the resulting stopped-flow traces were fit to a single-exponential process with eq 1. The observed rate constants *k*_obs_ were hyperbolically dependent upon the d-leucine concentration (Figures 2–4), allowing for the determination of the limiting rate constant for flavin reduction *k*_red_ (Table 2). The kinetic data for the observed rate of flavin reduction for all of the variant enzymes were fit with eq 2 (Figures 2–4), with *k*_red_ values similar to *k*_cat_ values (Table 2), consistent with hydride transfer being rate limiting with d-leucine as a substrate. The apparent equilibrium constant for substrate dissociation from the Michaelis complex *K*_d_ could be determined for all variant enzymes with d-leucine (Table 2). There was a 2-fold decrease in the *k*_red_ values for all of the variant enzymes compared to the wild-type enzyme. In contrast, the *K*_d_ values for all variant enzymes increased by 2-fold compared to the wild-type enzyme, as shown in Table 2. d-Leucine was used as a substrate instead of d-arginine because, at 25 °C, more than 80% of the rate of flavin reduction with the physiological substrate d-arginine occurs in the mixing time of the stopped flow (i.e., 2.2 ms) as in the case of the wild-type enzyme.^42,46^

## Discussion

This study aimed to investigate whether selectively increasing the rate of product release without significantly altering catalysis could translate into an increased rate of enzyme turnover in *Pa*DADH. From the X-ray crystallographic data of *Pa*DADH, the loop L2 E^246^ residue is found at the active site entrance and interacts with both the active site gate Y^53^ and the iminoarginine product guanidinium group (Figure 1). Thus, E^246^ was proposed to optimize d-arginine binding by ensuring a closed active site upon substrate capture and securing the iminoarginine product through interactions with the guanidinium group. From the site-directed mutagenesis, steady-state, and rapid-reaction kinetic data presented in this study, the E^246^ residue has been identified to interact with the guanidinium group of the iminoarginine product. This interaction dictates the rate of iminoarginine product release, resulting in faster enzyme turnover upon mutation of E^246^. Evidence to support the conclusions is discussed below.

### E^246^ Mutation Increases the Rate of *Pa*DADH Turnover with d-Arginine

This conclusion is supported by the steady-state and rapid-reaction kinetic data with the *Pa*DADH variant and wild-type enzymes at pH 10.0 and 25 °C. With d-arginine, there was a 4-fold increase in the *k*_cat_ value from 122 to 500 s^–1^ when the E^246^ residue was mutated to glycine in the E^246^G variant. Similarly, there was a ∼2-fold observed increase in the *k*_cat_ values for the E^246^L and E^246^Q variant enzymes (Table 1). The data are consistent with an increased rate of enzymatic turnover, resulting from mutation of the E^246^ residue, irrespective of the identity of the amino acid at position 246. Previous steady-state and kinetic solvent viscosity studies on *Pa*DADH established that both hydride transfer and the iminoarginine product release are partially rate-limiting for the overall turnover *k*_cat_ of the enzyme with d-arginine.^42^ Considering that, with all variant enzymes, flavin reduction occurred as rapidly as previously established for *Pa*DADH wild type,^42,46^ the effect of the mutation on the hydride transfer step with d-arginine can be considered negligible. Thus, the increased rate of *Pa*DADH turnover with d-arginine upon E^246^ mutation is explained by an increased rate of iminoarginine product release from the active site of the enzyme as a result of the loss of the 3.7 Å direct or water-mediated E^246^–guanidinium interaction that holds the product after catalysis (Figure 1), resulting in a faster rate of iminoarginine release. With the E^246^G variant, the additional 2-fold increase can be explained as the complete removal of the side chain at position 246, creating a wider active site entrance that allows for an even faster rate of product release.^41,44^

### E^246^ Mutation Has a Small Effect on the Overall Catalysis of *Pa*DADH with d-Leucine

Evidence for this conclusion comes from the steady-state and reductive half-reaction of *Pa*DADH E^246^Q, E^246^G, E^246^L, and wild-type enzymes with d-leucine at pH 10.0 and 25 °C (Table 2). d-Leucine was tested as a control substrate as a result of the inability of its side chain to interact with the residue at position 246 in *Pa*DADH. The rapid-reaction kinetics showed a 2-fold decrease in the hydride transfer rate *k*_red_ and a 2-fold increase in the *K*_d_ value for all variant enzymes compared to the wild-type *Pa*DADH (Table 2). The decreased rate of hydride transfer might be due to a different configuration or a decreased probability of the enzyme–substrate (ES) complex competent for the hydride transfer reaction upon mutation of the E^246^ residue. The increased *K*_d_ values are expected for mutating a loop residue that controls the active site gate because the removal of the residue renders the active site more open,^20^ leading to increased rates of substrate dissociation. The increased rate of substrate dissociation is picked up by the 2–4-fold lower second-order rate constant *k*_cat_/*K*_m_, which measures the ability of the enzyme to bind the free substrate to form the enzyme–substrate complex that undergoes catalysis,^21^ as observed for both the d-leucine and d-arginine substrates (Tables 1 and 2). Upon E^246^ replacement with glutamine, glycine, or leucine, the hydrogen bond interactions with the Y^53^ gate of loop L1 are hindered, likely leading to altered loop motions as recently reported for the *P. aeruginosa* NADH:quinone oxidoreductase and pyranose 2-oxidase when the Q^80^ and F^454^ gating loop residues were respectively mutated.^20,50^

**Scheme 2:**
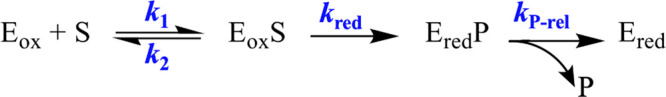
Reductive Half-Reaction Pathway of the *Pa*DADH E^246^ Variant Enzymes

For the E^246^G variant enzyme with d-leucine as a substrate, using eq 3, a rate constant for the iminoleucine release *k*_P-rel_ of ∼188 ± 7 s^–1^ could be estimated from the *k*_red_ and *k*_cat_ values of the enzyme (Table 2). Such a rate is only ∼4× faster than the rate of catalysis for the enzyme, consistent with both hydride transfer and product release contributing toward the overall turnover of the enzyme.^51,52^ Similarly, when the *k*_P-rel_ values were computed for the E^246^Q, E^246^L, and wild-type enzymes, the estimated rates for iminoleucine release were only ∼4 times faster than the rates of catalysis, suggesting that, with d-leucine, both the rate of hydride transfer and the rate of product release contribute toward the overall turnover approximately to the same extent for all enzymes. A likely reason for this observation is that, regardless of the amino acid residue at position 246 in *Pa*DADH, the rate-limiting steps during enzyme catalysis are comparably affected by the probability of the active site gating loop to exist in either the open or closed conformations. This reasoning implies that, irrespective of the loop dynamics and the mutation, the hydride transfer and product release steps are not altered to the extent that perturbs their overall contributions to *Pa*DADH turnover with d-leucine. Additionally, from the observed zero *y* intercepts from the best fit of the hyperbolic dependence of the observed rate of flavin reduction *k*_obs_ as a function of the d-leucine concentration for all enzyme variants (Figures 2–4), flavin reduction is irreversible for d-leucine conversion to iminoleucine in *Pa*DADH as reported for the wild-type enzyme,^45,46^ irrespective of the mutation at position 246 (Scheme 2). Thus, the replacement of E^246^ did not significantly alter the overall catalysis of *Pa*DADH.
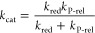
3

## Conclusion

The success of the E^246^ mutation in increasing the rate of *Pa*DADH turnover with d-arginine is a prime example of how deconstructing catalytic processes and targeting specific steps in the catalytic cycle of an enzyme can be explored for the bioengineering of biocatalysts. An important implication of this study is that we have engineered a d-amino acid oxidizing enzyme that can detect and consume its substrate faster at 500 s^–1^, providing a highly efficient system for applications in the food and l-amino acid industries.^5−8^ With the surging interest in gating residues as targets for protein engineering toward therapeutic development, it is vital to identify the properties of gating residues that alter enzyme activity and specificity.^9,27−29,34,53−58^ Given that gates control the flux of substances in and out of the active site,^9,41,59−63^ and because most gating residues are distal from the active site, their mutations tend to alter substrate selectivity and not enzyme catalysis.^41,44,55,63,64^ This study demonstrates that the proximity of a gating residue to the active site and interactions with ligands are two important factors to consider during protein engineering. Residues like E^246^ of *Pa*DADH, which is close to the active site and interacts with a substrate or product, could be prime targets for engineering faster biological catalysts. With more studies on flexible gating loops, there can be better informed protein engineering targets for therapeutic design, even for the *Pa*DADH host bacterium *P. aeruginosa*, a high-risk human pathogen notorious for developing antibiotic resistance.^65−73^

In summary, site-directed mutagenesis, steady-state, and rapid reaction kinetics have been used to generate the E^246^Q, E^246^G, and E^246^L variant enzymes of *Pa*DADH to investigate how improving the rate of product release could translate into an increased rate of enzyme turnover in *Pa*DADH. The specific role of the E^246^ residue of loop L2 in *Pa*DADH catalysis has been explored. From the study, E^246^ has been identified to participate in both substrate capture and catalysis in *Pa*DADH. With d-arginine, the E^246^ residue controls the rate of the iminoarginine product release through its interaction with the guanidium group, resulting in a faster rate of enzyme turnover upon E^246^ mutation.

## Abbreviations Used

*Pa*DADH: *Pseudomonas aeruginosa*d-arginine dehydrogenase
E^246^Q: glutamate 246 to glutamine mutant
E^246^G: glutamate 246 to glycine mutant
E^246^L: glutamate 246 to leucine mutant
